# Does stopping left-right nodal flow mirror impaired mechanosensation in the left-right organizer?

**DOI:** 10.1186/2046-2530-4-S1-P39

**Published:** 2015-07-13

**Authors:** R Jacinto, P Sampaio, M Roxo-Rosa, SS Lopes

**Affiliations:** 1 Chronic Diseases Research Center, CEDOC, NOVA Medical School / Faculdade de Ciências Médicas, Universidade Nova de Lisboa, Campo Dos Mártires Da Pátria, 130, 1169-056 Lisboa, Portugal

## Background

In most vertebrates organ asymmetries arise in early development through the left-right organizer (LRO). LRO ciliated cells induce a leftward fluid flow responsible for the asymmetric expression of Nodal pathway genes in tissues that will originate internal organs. We have recently published that *charon*/*dand5 *transcription is an early flow target in zebrafish by being expressed in the LRO, first symmetrically and later asymmetrically to the right where the fluid flow is weaker. We showed that absence of flow originates symmetric *charon *expression [1]. Pkd2 has been reported as part of a mechanosensor complex that senses flow and induces a calcium inward flux in kidney cells [2] and mouse LRO [3]. In agreement, mouse and zebrafish Pkd2 mutants have LR defects [4,5].

## Objective

Determine if impaired mechanosensing renders the same *charon*/*dand5 *phenotype as absent flow.

## Methods

We injected two morpholinos in zebrafish embryos: a dnah7-MO to stop cilia movement [1] and a pkd2-MO. We also co-injected both. We recorded flow by particle analysis, quantified Pkd2 expression and correlated these with *charon*/*dand5 *expression patterns by *in situ *hybridization and qRT-PCR.

## Results

50-60% of injected embryos have symmetric *charon*/*dand5 *expression when flow is low and homogeneous or when Pkd2 is absent from LRO ciliary membrane. We will show if *charon/dand5 *quantification by qRT-PCR is also similar. When both flow and Pkd2 are impaired, 80% of embryos showed symmetric *charon*/*dand5*, which indicates a synergistic effect.

## Conclusion

Although no flow and no Pkd2 have virtually the same phenotype, there seems to be a positive epistasis when both are affected.

## Acknowlegements

Supported by FCT-ANR/BEX-BID/0153/2012 grant and PD/BD/52420/2013 PhD scholarship.
